# Assessment of status of solid waste management in Asella town, Ethiopia

**DOI:** 10.1186/s12889-019-7551-1

**Published:** 2019-09-12

**Authors:** Gorfnesh Lema, Million Getachew Mesfun, Amade Eshete, Gizachew Abdeta

**Affiliations:** 1 Hirsch Institute of tropical medicine, Arsi University College of health science, Asela, Oromia Ethiopia; 2 Department of medical laboratory technology, Arsi University College of health science, Asela, Oromia Ethiopia; 3 Department of public health, Arsi University College of health science, Asela, Oromia Ethiopia

**Keywords:** Solid waste management, Asella, Ethiopia

## Abstract

**Background:**

Improper solid waste management (SWM) is a major public health and environmental concern in the urban areas of many developing countries such as Asella Town. The aim of this study was to assess the status of SWM in Asella town.

**Methods:**

A community-based cross-sectional study design was used to assess the status of improper SWM and associated factors in Asella town. From the total of eight kebeles (smallest administrative unit in Ethiopia) four kebeles were randomly selected using lottery method. The sample size was 413 households. The households were proportionally allocated to each randomly selected kebeles. The data was collected by pretested questionnaire in the local language. Data was entered using statistical software Epi Info version seven and transferred to SPSS version 21. Descriptive data analysis was done to summarize the socioeconomic status of the respondents. Chi-square was used to show the association between the status of solid waste management and different variables. Binary logistic regression was used to determine the potential factors for improper SWM.

**Result:**

332 (82.8%), had improper solid waste management practice. Lack of adequate knowledge about solid waste management and not having access to door to door solid waste collection could have contributed to the reported improper solid waste practice. Participants who didn’t have access to door to door solid waste collection service were about three times more likely to practice improper solid waste management when compared to those who had access (AOR = 2.873, 95 CI (1.565,5.273) *P* = 0.001).

**Conclusion:**

The study finding showed that, the majority of the residents practiced improper solid waste management. Lack of adequate knowledge about solid waste management and not having access to door to door solid waste collection could have contributed to the reported improper solid waste practice. Therefore, there is a need to enhance the awareness of the community about proper SWM and to improve the door to door solid waste collection service by the town municipality.

**Electronic supplementary material:**

The online version of this article (10.1186/s12889-019-7551-1) contains supplementary material, which is available to authorized users.

## Background

Solid waste management (SWM) in an urban area is a complex activity that involves the collection, transportation, recycling, resource recovery and disposal of solid waste generated in an urban area [1]. Municipal solid waste is composed of different wastes generated by households and different institutions such as schools, hospitals, slaughter houses and public toilets [2].

Municipal wastes are not well managed in developing countries due to the alarmingly increasing solid waste production which is more than the capacity of the cities and municipalities. It was reported that, waste collection rates are often lower than 70% in low-income countries and more than 50% of the collected waste is often disposed of through uncontrolled land filling [3].

Ethiopia is one of the low income countries facing the consequence of improper solid waste management. It was reported that about 20 to 30% of the waste generated in Addis Ababa, the capital city, remains uncollected [4]. Proper solid waste management requires the commitment of the town municipality and the active involvement of the community members. There are many initiatives taking place in Ethiopia to improve the environmental health especially in the capital city. In Addis Ababa the awareness of the community members about solid waste management is enhanced and more than 70% of the community inhabitant were willing to pay for door to door solid waste collection service which is one of the initiatives introduced by the government [5].

Asella is an old town with many public and private hospitals, health centers, industries, hotels and small scale enterprise where lots of solid waste is generated. The town municipality is mainly responsible for solid waste management of the town as there is no private organization involved in such tasks. There is no communal solid waste container deployed in different sites of the town, as a result solid waste produced from every household are collected on road side. Even though, solid waste management is supposed to be one of the critical public problems in Asella, there was no study done to systematically asses the magnitude of the problem and the factors for the improper waste management. The aim of this cross sectional study was to assess the status of household based improper solid waste management and to identify factors for improper solid waste management in Asella town. Findings of the study could help local policy makers to develop solid waste management related problems solving policies. It could also serve as baseline study to conduct similar study at a regional level.

## Methods

### Study area, design and subjects

The study area, Asella Town, is a capital city of Arsi zone established in 1945. It is located 175 Kmsoutheast of Addis Ababa. The total area of Asella town is about 4623 ha. Topography is characterized as rugged and inclined. The city is mainly characterized as highland’s climate condition. According to the census conducted by Central Statistical Agency (CSA) in 2007, the total population of Asella city was 65,250 with a growth rate of 2.99%. The major economic activities in the city are trade, urban agriculture, investment such as hotel, construction, factories, flour and food complex, food oil production, micro and small-scale enterprises and other informal business activities like street trades [6]. In Asella town, open dumping is the common method of waste disposal practice. Almost all generated solid waste in this town is indiscriminately dumped into streams, open surfaces, ditches, and residential compounds and along the highway crossing the town.

A community-based cross-sectional quantitative study design was used to assess the status of solid waste management and associated risk factors in Asella town from March 1/ 2017 to June 20/2017.

The sample size was calculated using computer generating (Epi Info) statCalc [7] considering the total estimated household number of the town, 15,966 at 95% level of confidence. Adding the 10% contingency the final sample size was 413.

From the total of 8 kebeles (smallest administrative unit in Ethiopia) 44 kebeles were randomly selected using lottery method. The calculated household number was proportionally allocated to each randomly selected kebele.

### Operational definitions

Improper solid waste management; in this study improper solid waste management practice is defined as not separating solid waste appropriately and/or disposing solid waste at a legally unauthorized place.

### Data collection procedures

The data including sex, age, educational status, marital status, family size and practice of solid waste management were collected through an interviewer and self-administered pretested and updated questionnaires with both open and close ended questions (Additional file 1). The questionnaire was adopted from different guidelines and customized according to the study area setup. It was initially prepared in English and translated to the local language, Afan Oromo. The questionnaire was tested and edited before the actual data collection. Field observation was employed for understanding households’ solid waste management condition, dumping in open space, river ditch and road, solid waste collection and transportation systems and disposal site facilities of the town. Photographs were taken during field observation for dumping sites and illegal solid waste management practice by the community. Data collection and analysis were done according to STROBE (Strengthening The Reporting of Observational Studies in Epidemiology) checklist.

### Data processing and analysis

The data was entered using statistical software Epi Info version seven and transferred to SPSS version 21. Descriptive data analysis was done to summarize the socioeconomic status of the respondents. Chi-square was used to show the association between the status of solid waste management and different variables. Binary logistic regression was used to determine the best predictors of improper solid waste management.

### Limitation of the study

The term improper solid waste management is broad. Separation of waste is also relative as there is no standard regulation of solid waste separation practiced in the community.

### Ethical considerations

The study was ethically approved by the ethical review committee of Arsi University, College of Health Sciences Formal letters were written to all concerned authorities and permission was secured at all levels. As the study didn’t include any invasive procedures, only informed verbal consent was collected from each respondent after explaining the purpose and procedure of the study. Written consent was exempted by the research review committee of the college. The anonymity of participants was maintained.

## Results

From the randomly selected households, 401 were included in to the assessment. This makes the response rate 97%. 285 (71%) of the respondents were female. The mean age of the respondents was 38 (+SD of 11.97) and 166 (44.1%) were in the age range of 26–35 years. Regarding their marital status, 276(68.8%) and 59(14%) of them were married and single, respectively. Orthodox Christian was the predominant religion 257(63.9%) among the study participants. 306 (76.3%) of the participants earn monthly income less than 80 USD. (Table 1).

**Table 1:** Demographic characteristics of the respondents of Arsi Zone Oromia Regional State Asella town on MSWM in 2017

Variable	Frequency	Percent
Age Group		
15–25	39	9.7
26–35	166	41.4
36–45	109	27.2
Above 45	87	21.7
Gender		
Female	285	71
Male	116	29
Educational status		
Unable to read and write	34	8.5
Able to read and write	99	24.7
Grade 9–12 complete	177	44.1
Diploma	43	10.9
First degree and above	48	12
Occupational status		
Government employee	133	33.2
Merchant	71	17.7
Daily labour	47	11.7
Urban agriculture	15	3.7
Housewife	111	27.6
Others	24	6

### Composition of municipal solid waste in Asella

**Fig. 1:**
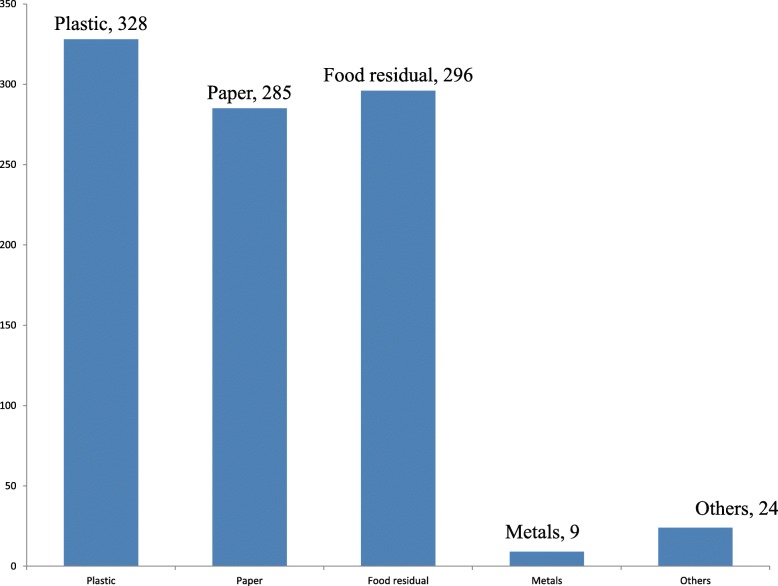
Frequency distribution of types of solid waste generated and disposed of by households from multiple responses in Arsi Zone Oromia Regional State Asella town, 2017

Variety of solid waste was reported to be generated from the households included in to the study. Accordingly; 328, 296 and 285 households’ heads have reported that they generated plastic, food residual and paper wastes, respectively (Fig. 1).

One hundred nine (27.2%) of households separated their solid waste at source. Solid waste was collected by the municipality in more than 1 month from 192(47.9%) households and 81(20.2%) of the households had no solid waste collection service. Majority, 359(89.5%) of the households did not practice reduce and reuse strategy and 307(76.6%) households burned solid waste in their compound (Table 2).

**Table 2:** Frequency distribution of households solid waste management practices in Arsi Zone Oromia Regional State Asella town, 2017

Variables	Frequency	Percentage
Separation solid waste at your home		
Yes	109	27.2
No	292	72.8
Collection interval of SW		
Once a two week	55	13.7
Once a month	51	12.7
More than a month	192	47.9
No serve at all	81	20.2
Practicing Reduce, Reuse and Recycle strategy		
Yes	42	10.5
No	359	89.5
Disposing SW on the road		
No	304	75.8
Yes	97	24.2
Do you dispose of SW in the ditch		
No	367	91.5
Yes	34	8.5
Do you dump SW in the yard		
No	372	92.8
Yes	29	7.2
Burn SW in the compound		
No	94	23.4
Yes	307	76.6

**Fig. 2:**
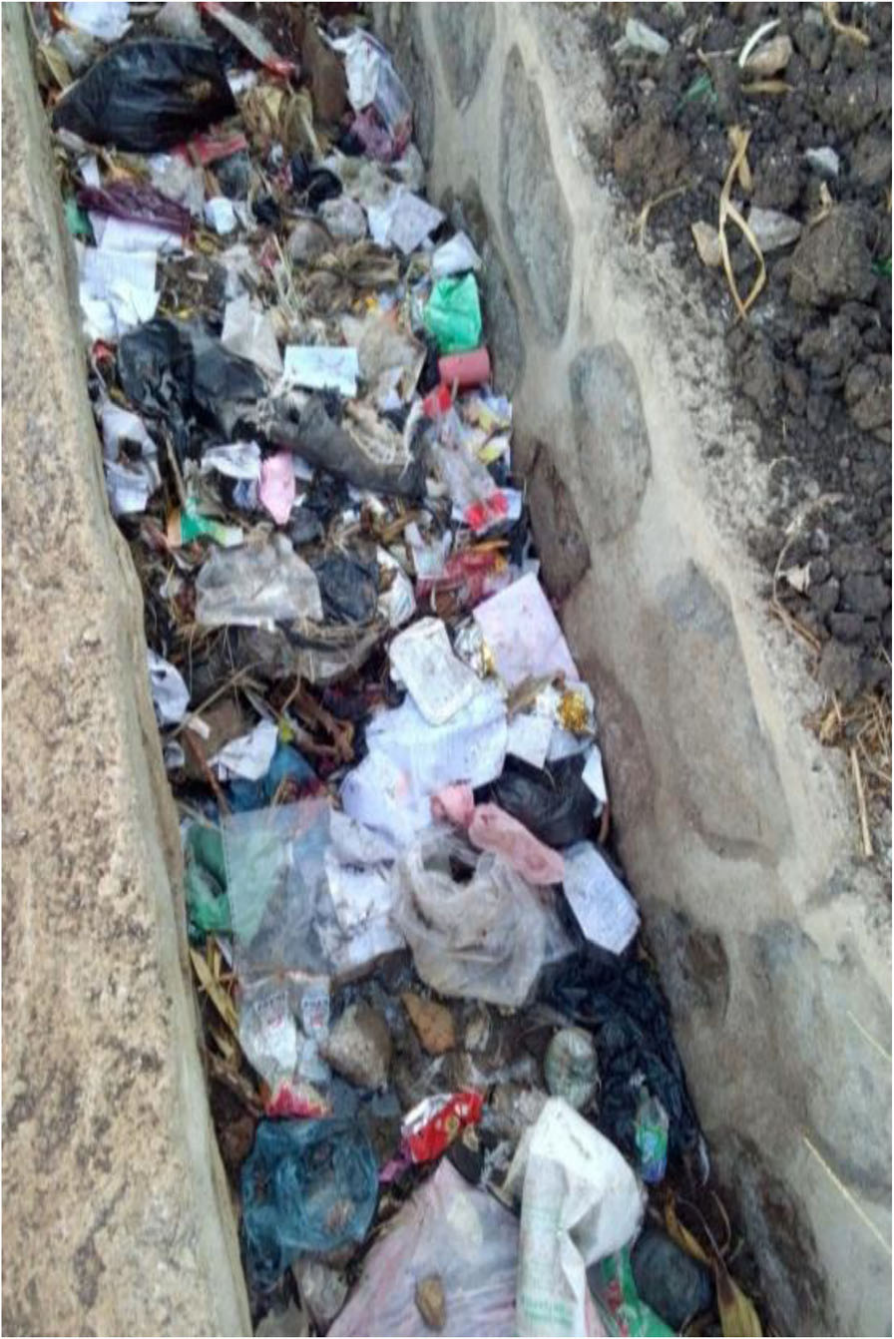
Waste disposed of in Ditches (Around Welkesa Kebele) of Asella town, 2017. (Image taken by the corresponding author)

**Fig. 3:**
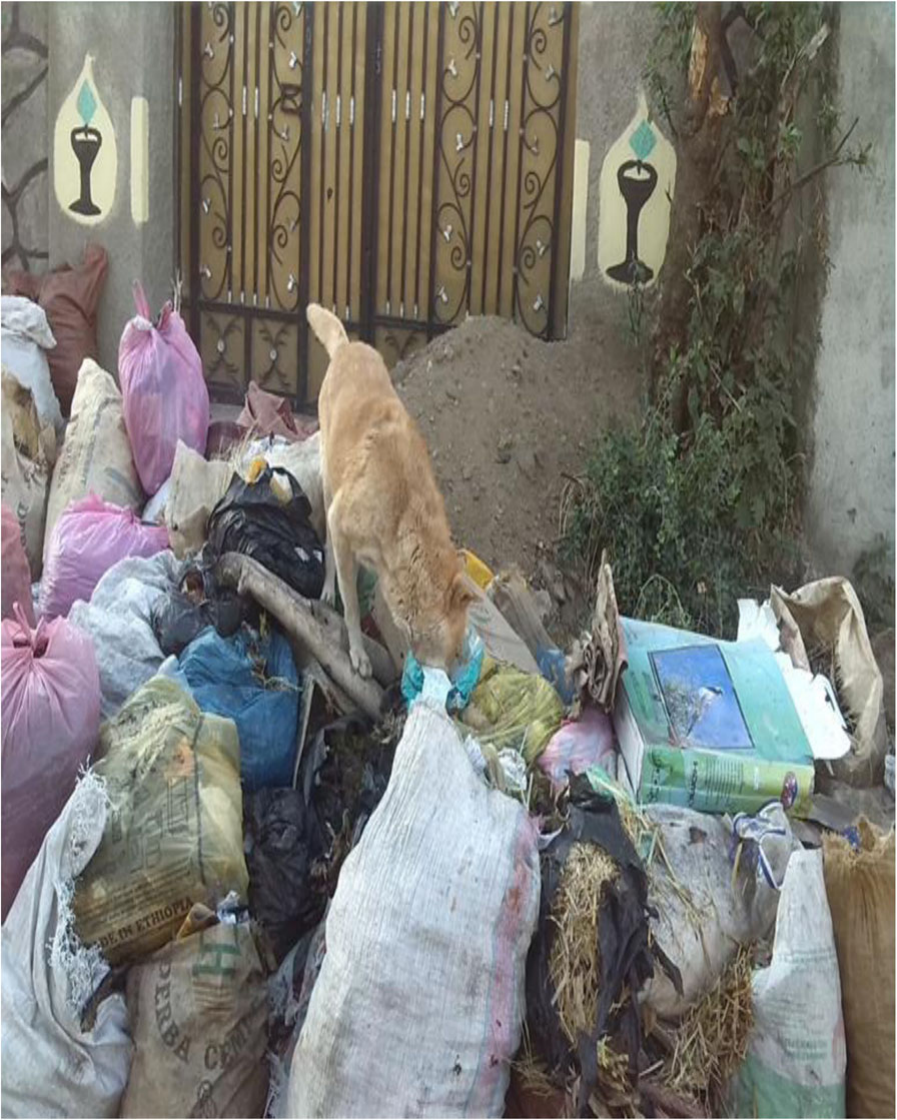
Illegal temporary storage (transfer station in front of residents’ house), Asella town 2017. (Image taken by the corresponding author)

332 (82.8%) participants had improper solid waste management practice (Figs. 2 and 3). Not having access to door to door solid waste collection service from municipality, not having knowledge about solid waste management and 3R (*P* = 0.001), and being unwilling to pay for solid waste management (*P* = 0.024) had statically significant association with improper solid waste management practice (Table 3). Other factors such as education status and occupation had no statistically significant association (*P* > 0.05) with improper solid waste management practice.

**Table 3:** Cross tabulation of contributing factors for improper solid waste management and association with the status of SWM in Arsi Zone Oromia Regional State Asella town, 2017

Variables		Solid waste management practice				*p* value
		Proper		Improper		
		N	%	N	%	
Gender	Female	46	16.2	238	83.8	0.467
	Male	23	19.7	94	80.3	
Family size	1–3	21	12.7	145	87.3	0.05
	4–6	45	21.6	163	21.6	
	7–9	3	11.1	24	88.9	
Access to door to door waste collection	Yes	49	28.7	122	77.3	< 0.001*
	No	20	8.7	210	91.3	
Knowledge of rule and regulation of SWM	Yes	16	37.2	27	62.8	0.001*
	No	53	14.8	305	85.2	
Knowledge of about 3R	Yes	24	30.8	54	69.2	0.001*
	No	45	13.1	278	86.1	
Willing to pay for SWM	Yes	65	19	278	81	0.024*
	No	4	6.9	54	93.1	
Consider waste as a resource	Yes	44	25	132	75	< 0.001*
	No	25	11.1	200	88.9	
Practicing 3R	Yes	17	40.5	25	59.5	< 0.001*
	No	52	14.5	307	85.5	

### Factors contributing to improper solid waste Management in Asella town

All the independent variables with *P*-Value less than 0.05 by bivariate analysis were entered into a multivariate logistic regression analysis, to analyse the contributing factors for improper solid waste management. Those households who had no door to door solid waste collection service were about three times more likely to practice improper solid waste management (Adjusted Odds Ratio(AOR) = 2.883, 95% Confidence interval(CI) (1.5705, 5.295) *P* = 0.001) than those who had the service (Table 4).

**Table 4:** Bivariate and multivariate analysis for factors association with improper solid waste management in Arsi Zone Oromia Regional State Asella town, 2017

Variables		Improper SWM		OR (95% CI)				
		Yes	No	COR	AOR	95% C.I		
No access to door to door solid waste collection	Yes	210	20	4.217	2.883	1.570	5.295*	0.001*
	No	122	49	1	1			
Inadequate knowledge of rule and regulation of SWM	Yes	305	53	3.41	2.029	0.966	4.260	0.062
	No	27	16	1	1			
Not considering waste as a resource	Yes	200	25	2.667	1.506	0.767	2.576	0.271
	No	132	44	1	1			
Inadequate knowledge of about 3R	Yes	278	45	2.746	1.623	0.693	2.735	0.361
	No	54	24	1	1			
Unwilling to pay for SWM	Yes	54	4	3.156	2.059	0.659	5.884	0.225
	No	278	65	1	1			

* Statistically significant associationn

## Discussion

The majority, 72.8%, of respondents didn’t separate solid waste at source. The type of wastes generated includes; Plastic (34.8%), Food residual (31.4%), Paper (30.3), Metal wastes (1%) and other wastes (2.5%). The high level of improper Solid waste management practice were consistent with study findings from Nigeria 83.3% [8], Ghana 82.7% [9] and Gonder, Ethiopia 69.7% [10]. The current solid waste management practice was much lower than the practice seen in Kampala [11]. Even though the economic status of the town is relatively similar with the study area, due to the fact that the people in Kampala use their solid waste to produce manure, their solid waste management practice was much higher than households in Asella town.

In developing countries improper solid waste disposal is common and about half of the respondents in our study dispose their solid waste improperly (dumping in the yard, burn in their compound, throwing in the ditch and in the river). A study done in Keko Machungwa, Tanzania revealed that 62% of residences dispose wastes in unauthorized place, this high improper solid waste disposal was because of inaccessibility due to informal settlements and narrow roads [12]. Similarly, a high improper solid waste disposal practice 75% was reported from Debrebrihan, Ethiopia [13].

This high improper solid waste disposal practice in our study area could be because of the long door to door solid waste collection interval, in which only 26% of the households had the service monthly. Lack of door to door solid waste collection service by town municipality was found to be the potential risk factor for improper solid waste management practice in Asella town, as those households with door to door solid waste collection service were about three times more likely to have proper solid waste management practice.

There was relatively low access to the door to door solid waste collection service and the long interval of collection compared to the study done in the capital city, Addis Ababa. In which 84% of the households had access to door to door solid waste collection [14]. This difference in the accessibility of door to door collection service could be due the poor infrastructure in Asella town and difference in regulations of solid waste management. In the capital city there is better infrastructure of transport in which most households are accessible and the service is accessible for those who are willing to pay [5, 14]. But in Asella town, households are not easily accessible due to the lack convenient roads and the door to door solid waste collection is handled mainly by the town municipality in which there is no direct payment for each solid waste collection service. Similar studies done in Mombasa, Kenya [15] and Adama, Ethiopia [16] showed that there were better door to door solid waste collection service.

Besides to the poor door to door solid waste collection service, the awareness of the households about rules and regulation of solid waste management (10.7%) and practice of 3R (10.4%) were poor. The awareness of solid waste management in the study area was lower than the study findings from Gonder [10] and Bahirdar, Ethiopia [17]. This difference in awareness could be due to the involvement of Nongovernmental organizations such as the Dream Light private limited company in Bahridar which is mainly engaged in proper solid waste management awareness creation among the community members of the city [17].

## Conclusion and recommendations

This study showed that, more than 82% of Asella town residents practice improper solid waste management. Lack of adequate knowledge about solid waste management and not having access to door to door solid waste collection could have contributed to the reported improper solid waste practice. Therefore, there is a need for enhancing the awareness of solid waste management at the community level. We also recommend the municipality of Asella to enhance the accessibility of door to door solid waste collection service. Further studies to assess the status of SWM in different town within the district could help to have comprehensive image about SWM in the district and to convince policy makers to give emphasis to the issue.

## Additional files

Additional file 1: Questionnaire prepared for households. (DOCX 17 kb)

## Data Availability

The datasets analysed during the current study are available from the corresponding author on reasonable request.

## Abbreviations

AOR: Adjusted Odds Ratio
CI: Confidence interval
CSA: Central Statistical Agency
SWM: Solid waste management
