# Supplementary material for: Assessment of status of solid waste management in Asella town, Ethiopia
Source: BMC Public Health. 2019 Sep 12;19:1261. doi: 10.1186/s12889-019-7551-1 (PMC6739914; doi:10.1186/s12889-019-7551-1)
Supplement: Supplementary file 1 — Questionnaire prepared for households. (DOCX 17 kb) [file 12889_2019_7551_MOESM1_ESM.docx]

: Questionnaire prepared for households.

I Socio-Demographic information of households.

| Code  No | Statements | Response: Circle your choice or write your opinion. |  |
| --- | --- | --- | --- |
| Q101 | 1. Kebele | 1.Arada 2. Wakessa  3.Chilalo 4. Konbolcha |  |
| Q102 | Gender | 1. Female 2. Male |  |
| Q103 | Age in year | ___________________ |  |
| Q104 | Marital Status | 1.Married 2.Single  3.Divorced 4. Widowed |  |
| Q105 | Religion | 1.Orthodox 2.Muslim  3.Protestant 4.Catholic 5. Others |  |
| Q106 | Educational status | 1.Can not read and write  2. Can read and write  3.Grade 9-12 4.Diploma  5.First degree and above |  |
| Q107 | Occupation | 1.Employee 2. Merchant  3.Daily laborer 4. Urban agriculture  5.House wife6.Other(specify) |  |
| Q108 | Family size | _______________ |  |
| Q109 | Household income | 1.<500 Birr 2.501-1000 Birr 3.1001-1500 Birr 4.1501-2000 Birr 5. Above 2001 |  |

II. Questionnaires prepared for households to identify practices of solid waste management.

| No | Statement | Response; circle or give your opinion |  |
| --- | --- | --- | --- |
|  |  |  | Remark |
| Q201 | Do you separate solid waste at house level | 1.No  2.Yes |  |
| Q202 | How many materials do you use for storages of waste produce in your dwellings | 1.One  2.Two  3.Three |  |
| Q203 | How much kg Solid waste do you generate per week? | ________________ |  |
| Q204 | Types of waste generate/composition of waste | 1.Plastic 2.food residual 3.metals  4.Papers 5.Fruit and vegetable residuals 6.Others |  |
| Q205 | From your family who is responsible for waste collection and disposal | 1.Female 2.male 3.Both |  |
| Q206 | Access door to door collection | 1. No 2. Yes |  |
| Q207 | Is collection service in the town good? | 1. No 2. yes |  |
| Q208 | Streets keep clean in your hometown. | 1. No 2. Yes |  |
| Q209 | Do you know rule and regulation of SWM | 1. No 2. Yes |  |
| Q210 | Have you ever heard 3R(Reduce, Reuse and Recycle | 1. No 2. Yes |  |
| Q211 | Do you practice 3R( Reduce, Reuse and Recycle the waste | 1. No 2. Yes |  |
| Q212 | Participation of community on waste management practices | 1.Poor  2.Good |  |
| Q213 | Where do you dispose waste to generate from your home | 1.On the road  2.At the temporary disposal site  3.At Riverside  4.Burn at compound  5.In the ditch  6.Dumping the yard |  |
| Q214 | Does municipal have good collection site? | 1. No 2. Yes |  |
| Q215 | Collection interval of municipal | 1.Per day  2.Per week  3.Per two week  4.Per month  5. Above one month  6.No service at all |  |
| Q216 | Willing to pay for waste management | 1.No 2. Yes |  |
| Q217 | How much money do you pay for waste collection per month | 1_______ |  |
| Q218 | Improper waste management causes health problem | 1.Strongly disagree  2.Disagree  3.Agree  4.Strongly agree |  |
| Q219 | Improper waste management causes environmental problem | 1.Strongly disagree  2.Disagree  3.Agree  4.Strongly agree |  |

III. Questionnaires prepared for households to evaluate factors contributing improper solid waste management in Assela town.

| CodeNo | Statement |  |  | | |
| --- | --- | --- | --- | --- | --- |
|  |  | Response .Circle or write your opinion | |  |  |
| Q301 | Does the municipality have good disposal site | 1. No 2. Yes | |  |  |
| Q302 | Municipal has good infrastructure for solid waste management like vehicles and human power | 1. No 2. Yes | |  |  |
| Q303 | Does the municipality prepare temporary storage in the town | 1. No 2. Yes | |  |  |
| Q304 | Does stakeholder involve in waste collection | 0. unknown  1. No 2. Yes | |  |  |
| Q305 | Do you get training about solid waste management by municipality | 1. No 2. Yes | |  |  |
| Q306 | Do you know how solid waste is managed? | 1. No 2. Yes | |  |  |
| Q307 | Does the municipality allocate enough budget for solid waste management in the town? | 1. No 2. Yes | |  |  |
| Q308 | Do you know waste is wealth? | 1. No 2. Yes | |  |  |
| Q309 | What are the challenges of municipal for proper solid waste management? | 1. Distance of temporary collection sites  2.Lack of monitoring and evaluation.  3. The absence of responsible person 4.Absence of the regular plan for collection.  5. Others(specify) | |  |  |
